# Exploring Tetrazolium Salt Reduction by Mono- and Bimetallic Nanoparticles as an Alternative Signal-Generation Strategy for Point-of-Care Diagnostics

**DOI:** 10.3390/bios16070360

**Published:** 2026-06-29

**Authors:** Paweł Stańczak, Maciej Trzaskowski, Mariusz Pietrzak

**Affiliations:** 1 Department of Medical Diagnostics, Centre for Advanced Materials and Technologies CEZAMAT, Poleczki 19, 02-822 Warsaw, Poland; pawel.stanczak2.dokt@pw.edu.pl; 2 Chair of Medical Biotechnology, Faculty of Chemistry, Warsaw University of Technology, Noakowskiego 3, 00-664 Warsaw, Poland; maciej.trzaskowski@pw.edu.pl

**Keywords:** nanozymes, nanoparticles, reduction, tetrazole salts, PoC, LFA

## Abstract

Nanozymes, nanomaterials that mimic enzymatic activity, offer superior stability, tunability, and lower production costs compared to natural enzymes. To date, most nanozyme-based point-of-care (PoC) diagnostic systems have relied on oxidation reactions, such as oxidation of 3,3′,5,5′-tetramethylbenzidine, which often suffer from limited substrate stability and high background signal. This study investigates reduction reactions, particularly those involving tetrazolium salts, as an alternative route for signal generation in PoC devices. For this purpose, monometallic and bimetallic gold, palladium, and platinum nanoparticles were synthesized via chemical reduction using poly(vinyl alcohol) as a stabilizing agent. The resulting nanoparticles were uniform in size and morphology. Their catalytic performance was confirmed through the reduction of 4-nitrophenol. The tetrazole salts were selected as promising substrates for application in PoC settings and further explored by examining the nanozyme-based reduction of 3-(4,5-dimethyl-2-thiazolyl)-2,5-diphenyl-2H-tetrazolium bromide (MTT). The nanozymes catalyzed the reduction of MTT in the presence of sodium borohydride, producing a distinct colorimetric signal under selected conditions. The effects of reducing agent concentration, buffer pH, and potential interferents were evaluated, with performance suitable for PoC devices achieved at basic pH and low borohydride concentration. Interference studies showed negligible MTT reduction in the presence of physiological levels of ascorbic acid, human serum albumin, and 10% concentration of human serum. Finally, a proof-of-concept lateral flow assay demonstrated successful signal generation through nanozyme-catalyzed MTT reduction. Results establish tetrazolium salts as suitable substrates for nanozyme-enhanced PoC diagnostics and highlight reduction-based chromogenic systems as a viable alternative to traditional oxidation-based assays.

## 1. Introduction

Nanozymes are nanomaterials that exhibit enzyme-like catalytic activity while offering several advantages over their natural counterparts, including improved stability under a broad range of pH, temperature, and ionic strength conditions, lower production costs, and readily tunable physicochemical properties [1,2]. Their high surface-to-volume ratio and size-dependent properties enable precise control of catalytic performance through modifications in composition, morphology, and surface chemistry [3,4]. In addition, the use of polymeric stabilizers during synthesis facilitates the functionalization of nanoparticles with biomolecules, such as antibodies and aptamers, making nanozymes particularly attractive for biosensing applications.

Since the term “nanozyme” was introduced by Scrimin et al. in 2004 [5], nanozymes have been extensively investigated for applications in medicine, environmental remediation, industrial catalysis, and diagnostics. Among these areas, point-of-care (PoC) diagnostics has emerged as one of the most promising fields due to the ability of nanozymes to enhance analytical signals and improve assay sensitivity [6,7]. Nanozymes that mimic glucose oxidase activity have been investigated for incorporation into wearable biosensors for continuous glucose monitoring. In contrast, horseradish peroxidase-like nanozymes are frequently employed in point-of-care (PoC) diagnostic platforms, including ELISA-based immunoassays such as lateral flow assays, microfluidic devices, paper-based analytical systems, and other miniaturized detection formats [8,9,10]. Since ELISA tests are regarded as the gold standard in enzyme immunoassays, many of their strategies have been translated into PoC tools utilizing nanozymes. Among these, the choice of substrate and its corresponding catalytic activity have become critical design choices. For example, 3,3′,5,5′-tetramethylbenzidine (TMB) is widely used as the model substrate for evaluating peroxidase-like activity of nanozymes [11]. Consequently, most diagnostic applications of nanozymes to date have primarily focused on oxidation reactions [12].

Despite their widespread use, oxidation-based nanozyme systems face several limitations that hinder their implementation in PoC devices. Many require high concentrations of oxidizing agents, which may degrade or passivate nanoparticle surfaces, thereby reducing catalytic activity [13,14]. In addition, commonly used substrates often require acidic conditions that are incompatible with physiological samples and user-friendly PoC workflows [15,16]. The spontaneous oxidation of substrates by atmospheric oxygen, instability of oxidants such as hydrogen peroxide, and the potential generation of reactive oxygen species (ROS) can further increase background signals and compromise assay reliability [17,18,19,20,21,22,23]. These issues frequently necessitate additional sample handling or storage, which is undesirable in decentralized diagnostic settings. In contrast to ROS, which are strong oxidants and can be generated in multiple ways in oxygen-rich environments, reducing agents are not formed as spontaneously in the natural environment (outside living organisms). This distinction highlights the potential advantages of developing nanozymes that mimic catalytic activity in reduction reactions for biosensing applications.

In contrast, nanozymes catalyzing reduction reactions remain largely unexplored for diagnostic applications. Existing studies have primarily focused on environmental remediation and industrial catalysis, particularly the reduction of nitroaromatic compounds such as 4-nitrophenol (4-NP), which serves as a standard model reaction for evaluating reductase-like nanozyme activity [24,25,26,27,28,29]. Additionally, some nanoparticles are explored as catalysts for hydrogen production in emerging sustainable energy systems [30,31]. Biomedical applications of nanozymes catalyzing reduction reactions are largely limited to medical and pharmaceutical fields, for example, in wound treatment, where their ability to scavenge reactive oxygen species has been explored [32,33]. In contrast to nanozymes, which exhibit oxidase- and peroxidase-like activity, only a few studies have reported diagnostic approaches that utilize reduction reactions to generate the analytical signal, most of which were published within the past few years. These studies focus on gold nanoparticles as nanozymes catalyzing the reduction of 4-nitrophenol, often employing aptamers as recognition elements. For instance, a recent study by Z. Khoshbin et al. reported an aptasensor for detecting food toxins, specifically acrylamide, using citrate-stabilized gold nanoparticles (AuNPs). They employed AuNPs as nitroreductase-like nanozymes, which could be trapped by oligonucleotides immobilized on a plastic sheet. These immobilized strands were complementary to another aptamer specific for acrylamide binding. When acrylamide was present, the specific aptamer detached from the immobilized strands due to its higher affinity for the analyte, resulting in the entrapment of AuNPs. After rinsing the plastic sheet, the gold nanoparticles catalyzed the reduction of 4-nitrophenol to 4-aminophenol, resulting in a color change in the sample buffer from yellow to clear, indicating the presence of the analyte [34]. Although this approach offers several advantages over substrates used with peroxidase-like nanozymes—such as a lower risk of nonspecific catalysis and easier handling of reducing agents in solid state (e.g., sodium borohydride or hydroquinone are stored as solids for commercial use)—it lacks the versatility and diversity of said substrates, which have been widely investigated over the years. For example, there is a choice of oxidase- or peroxidase-like substrates that can generate fluorescent (*o*-phenylenediamine [35,36], which can be also used as chromogenic substrate) or luminescent (e.g., luminol [37]) signals or form chromogenic precipitates upon oxidation (e.g., 3,3′-diaminobenzidine [38]), which is an essential property for substrates application in devices based on sample flow such as lateral flow assays or microfluidic platforms [39].

A major limitation of current reduction-based nanozyme diagnostics is the narrow range of available signal-generating substrates. In contrast to oxidation-based systems, which benefit from a diverse portfolio of chromogenic, fluorogenic, and precipitating substrates, few reduction-responsive substrates suitable for PoC applications have been investigated. Several tetrazolium salts exhibit physicochemical properties that are particularly advantageous for point-of-care (PoC) diagnostic applications. First, the formation of strongly absorbing or fluorescent formazan products enables highly sensitive colorimetric or fluorometric readouts without additional labeling or signal amplification. Second, depending on the molecular structure of the specific tetrazolium derivative, the resulting formazan products may be either soluble or insoluble in aqueous media. The formation of insoluble crystalline formazans is especially relevant for paper-based and flow-through diagnostic formats, as it allows spatial confinement of the signal at the site of the reaction, improving visual contrast and reducing signal diffusion. Third, many tetrazolium-based systems exhibit a low intrinsic background signal in their oxidized form, thereby enhancing signal-to-noise ratios upon reduction. Finally, the structural diversity of tetrazolium salts, including variations in aromatic substituents and charge distribution, enables tuning of their redox potential, spectral properties, and solubility, making them adaptable to different assay formats and detection strategies. For example, 3-(4,5-dimethyl-2-thiazolyl)-2,5-diphenyl-2H-tetrazolium bromide (MTT) is reduced from a yellow tetrazolium salt to dark blue formazan crystals, whereas 5-cyano-2,3-di-(p-tolyl)tetrazolium chloride (CTC) forms red fluorescent crystals with excitation and emission maxima at approximately 480 and 630 nm, respectively [40,41].

The present study addresses the lack of reduction-based signal-generation strategies for nanozyme-assisted PoC diagnostics by investigating the catalytic reduction of tetrazolium salts by metallic nanozymes. Palladium, gold, and platinum monometallic and bimetallic nanoparticles stabilized with poly(vinyl alcohol) (PVA) were synthesized and characterized in terms of their size and catalytic performance. Their ability to catalyze the reduction of tetrazolium salts was systematically evaluated and compared with the model reduction of 4-nitrophenol. To the best of our knowledge, this is the first study to demonstrate nanozyme-catalyzed tetrazolium salt reduction as a signal-generation mechanism for point-of-care diagnostics. By expanding the range of substrates available to reductase-like nanozymes, this work lays the foundation for developing new diagnostic platforms that overcome key limitations of conventional oxidation-based approaches.

## 2. Materials and Methods

### 2.1. Reagents

Chloroplatinic acid (8% (*w*/*v*) aqueous solution), sodium bicarbonate, sodium phosphate dibasic dihydrate, potassium phosphate monobasic, potassium nitrate, diethylenetriaminepentaacetic acid (DTPA), Tween-20, 4-nitrophenol, tetrazolium bromide, sodium caseinate, copper-supported carbon grid, human serum type AB, and human serum albumin, ethylenediaminetetraacetic acid (EDTA), N,N-Diethyl-p-phenylenediamine, 4-Hydroxy-1-naphthalenesulfonic acid sodium salt, Bilirubin, Heparin, Glucose, and Uric Acid were obtained from Sigma-Merck (Burlington, MA, USA). Sodium chloride and sodium borohydride were from Acros Organics (Geel, Belgium). Sodium hydroxide was obtained from Chempur (Piekary Śląskie, Poland). Bovine serum albumin was obtained from Glentham (Lincolnshire, UK). Tetrachloroauric (III) acid, potassium hexachloropalladate (IV), polyvinyl alcohol mean Mw 49 kDa, polyvinyl alcohol Mw 10–26 kDa 86–89% hydrolyzed, polyvinyl alcohol Mw 17.6–26.4 kDa 98–99% hydrolyzed, polyvinyl alcohol, Mw 57–66 kDa 86–89% hydrolyzed, 96-well MaxiSorp plate, and 96-well U-shaped plate were obtained from Thermo Scientific (Waltham, MA, USA). Human liver ferritin antigen, mouse anti-human ferritin antibodies, and goat anti-mouse antibody were purchased from MedixBiochemica (Espoo, Finland). All reagents were used as received. Unless otherwise stated, all solutions were prepared in ultrapure water DI.

### 2.2. Nanoparticle Synthesis

A 50 mM solution of sodium borohydride (NaBH_4_) was prepared in water. Solutions of poly(vinyl alcohol) (PVA) were prepared in 10 mM NaOH aqueous solution at a concentration of 1 mg mL^−1^. Precursor solutions (10 mM) were prepared in 10 mM NaOH aqueous solution using hydrogen tetrachloroaurate (III) trihydrate (HAuCl_4_ 3H_2_O), potassium hexachloropalladate K_2_(PdCl_6_), and an 8% aqueous solution of chloroplatinic acid (VI) (H_2_PtCl_6_). In a 2 mL Eppendorf-type microtube, the reagents were added in a strictly defined order: first, 1.2 mL of the stabilizer solution (PVA) was introduced, followed by 150 μL of the two precursor solutions mixed in a set volume ratio of palladium to gold and platinum for bimetallic nanoparticles and one precursor solution of the corresponding metal for monometallic NPs. Immediately afterward, 150 μL of the reducing agent (NaBH_4_) solution was added. The resulting reaction mixtures were placed on a shaker (Thermoshaker TS-100, BIOSAN, Riga, Latvia), covered with aluminum foil, and left under gentle agitation (480 RPM) for 7 days to allow nanoparticle growth processes inherent to the chemical reduction approach, including Ostwald ripening and coalescence, to complete.

### 2.3. DLS Analysis

Immediately before measurement, the nanoparticle suspension was sonicated in an ultrasonic bath to disperse any agglomerates that had formed. One milliliter of the nanoparticle suspension was transferred into a disposable polymer spectrophotometric cuvette and then placed in the Zetasizer Nano ZS analyzer (Malvern Panalytical, Malvern, UK). A new measurement protocol was created using the instrument’s dedicated software, with the measurement mode set to size determination, an incubation time of 30 s, and 11 measurement cycles, each lasting 10 s. Four measurement series were performed.

### 2.4. UV–Vis Spectroscopic Analysis of Nanoparticles

Immediately before measurement, the nanoparticle suspension was sonicated in an ultrasonic bath to disperse any agglomerates that had formed. One hundred microliters of nanoparticle suspension was mixed with the same amount of water and transferred into a U-shaped 96-well microplate, which was subsequently placed in the Multiscan FC microplate reader (Thermo Scientific). A new measurement protocol was created using the instrument’s dedicated software. The wavelengths were recorded over the range of 200 to 700 nm with a 1 nm increment for each nanoparticle suspension.

### 2.5. STEM Characterization of Size and Morphology

Bright-field STEM and STEM EDX images of the synthesized nanoparticles were acquired using a Hitachi SU8230 microscope (Hitachi High-Tech Corporation, Tokyo, Japan) operated at an accelerating voltage of 30.0 kV. Copper TEM grids coated with a Carbon film were used as sample supports. For the analysis, the nanoparticles were diluted 10 times in water. The grids were immersed in aqueous nanoparticle suspensions and air-dried prior to imaging.

### 2.6. Evaluation of 4-Nitrophenol Reduction

The substrate reaction solution was prepared in a Britton Robinson (0.04 M boric acid, 0.04 M phosphoric acid, and 0.04 M acetic acid) universal buffer, adjusted to the desired pH range, with final concentrations of 0.25 mM 4-nitrophenol and 10 mM NaBH_4_, and the measurements were carried out using a Tecan Sunrise microplate reader (Tecan, Männedorf, Switzerland). A U-shaped 96-well microplate was prepared by adding 150 μL of the substrate reaction mixture to each well in consecutive rows. In contrast, dilutions of the nanoparticle suspensions, prepared 10-fold in water, were added to the last row. The last column served as a negative control, in which the nanoparticle solution was replaced with deionized water. The measurement protocol, created in the instrument’s dedicated software, was set to a wavelength of 400 nm, kinetic mode, 30 cycles, with mixing between cycles. Immediately before initiating the measurement, 50 μL of each nanoparticle dilution from the last row was added to the substrate reaction mixture using a multichannel pipette, and the measurement was started immediately afterward. Data analysis was performed using the instrument’s software and Microsoft Excel, and the rate of absorbance change was determined from the slope of a linear fit to at least seven consecutive data points within the linear range of the kinetic curve, with each measurement performed in at least five replicates.

### 2.7. Evaluation of MTT Reduction

The substrate reaction solution was prepared in a universal buffer (adjusted to the desired pH range) with final concentrations of 2.0 mM MTT, NaBH_4_ in the range of 0.05–25.0 mM, and 10% (*v*/*v*) DMSO. The measurements were performed using the same procedure as for the 4-nitrophenol reduction assay, with nanoparticles diluted 200-fold. The measurement protocol, created in the instrument’s dedicated software, was modified to a wavelength of 570 nm.

### 2.8. UV–Vis Spectroscopic Analysis of MTT Reduction

The measurements were performed following the MTT reduction protocol, except that DMSO was added to the substrate solution before the reaction was initiated or after 10 min. Furthermore, the U-shaped 96-well microplate was placed in the Multiscan FC microplate reader instead of the Tecan Sunrise microplate reader. A new measurement protocol was created using the instrument’s dedicated software. The wavelengths were recorded over the range of 200 to 700 nm with a 1 nm increment for each nanoparticle suspension.

### 2.9. Investigation of Interferents’ Effect on Non-Catalytic Reduction of MTT

The study of the effects of interferents on the non-catalytic reduction followed the previously described MTT reduction protocol. The substrate solutions were prepared as follows: 50 mg mL^−1^ HSA or BSA, 20 μg mL^−1^ ascorbic acid, 2.2 mg mL^−1^ EDTA, 0.14 mg mL^−1^ heparin, 12.5 μg mL^−1^ bilirubin, 60 μg mL^−1^ uric acid, 1.0 mg mL^−1^ glucose, 10-fold-diluted human serum, and 200-fold-diluted nanoparticles, with 1 mM NaBH_4_ as a positive control and 1 mM NaBH_4_ alone as a negative control. Each solution also contained 2 mM MTT and 10% (*v*/*v*) DMSO in a universal buffer at pH 9.0. The measurements were performed following the same procedure as described for the MTT reduction assay.

### 2.10. Conjugation of Au:Pd Bimetallic Nanozymes with Mouse Anti-Ferritin Antibody

Conjugation was performed by the passive adsorption method through nanoparticle charge modification. In a 1.5 mL Eppendorf tube, 200 μL of phosphate buffer (100 mM, pH 7.0), 200 μL of Au:Pd nanoparticles (1:5 molar ratio, PVA 49 kDa), and 8 μL of monoclonal mouse anti-ferritin antibodies (5 mg mL^−1^) were combined. The mixture was gently stirred at 480 rpm on a shaker at 4 °C for 24 h. Subsequently, sodium azide was added to a final concentration of 0.05% to prevent microbial contamination. The resulting conjugates were stored in a sealed Eppendorf tube at 4 °C until further use.

### 2.11. NLISA Study on Conjugation Efficiency for Ferritin Detection

Testing of the conjugates using NLISA was performed on commercial 96-well MaxiSorp plates (Thermo Scientific) in both direct and sandwich assay formats. Monoclonal goat anti-mouse and monoclonal mouse anti-ferritin antibodies were diluted to 1.0 mg mL^−1^ in 50 mM carbonate buffer (pH 8.0). BSA solutions were prepared at 1.5% and 3.0% (%w) in PBS (137 mM NaCl, 2.7 mM KCl, 10 mM Na_2_HPO_4_, 1.8 mM KH_2_PO_4_, pH 7.4) containing 0.05% (%w) Tween 20 (PBST). Conjugates were diluted 80-fold in 1.5% BSA PBST. Ferritin antigen was diluted to 5 μg mL^−1^ in 1.5% BSA PBST. The substrate solution was prepared by diluting a 20 mM MTT DMSO solution 20-fold in Britton–Robinson universal buffer (pH 9.0) containing 1.0 mM NaBH_4_. Plates were initially washed with 50 μL of carbonate buffer, after which 60 μL of antibody solution or carbonate buffer was added per well. Plates were sealed with tape and a lid to prevent evaporation and incubated under gentle agitation (480 rpm, 4 °C, 24 h). The following day, the solution was discarded, and the wells were washed with 60 μL of 3% BSA in PBST. Plates were resealed and incubated on a shaker (480 rpm, 20 °C, 30 min). The conjugate solution, previously stored in an Eppendorf tube, was sonicated before use. After removing the blocking solution, 60 μL of conjugate solution was added to each well, and the plates were incubated on a shaker for 30 min. Following incubation, the conjugate solution was removed, and the wells were washed with PBST. Subsequently, 60 μL of ferritin solution (5 μg mL^−1^) was added to each well, and plates were incubated on a shaker for 30 min. After incubation, the solution was discarded, and the wells were washed three times with PBST. Finally, 150 μL of the MTT substrate mixture was added to each well, and the spectrophotometric measurement was performed according to the MTT reduction protocol (readout at 570 nm).

### 2.12. Lateral Flow Assay Development for Ferritin Detection

The lateral flow assays (LFA) were prepared in two independent repetitions conducted two weeks apart. A conjugate pad buffer composed of 10% sucrose, 0.5% Tween 20, and 2% (*w*/*w*) sodium caseinate in PBS (pH 7.4) was used to dilute the nanozyme–antibody conjugates 250-fold. In contrast, the control antibody solution was prepared by diluting rabbit anti-mouse antibodies from an initial concentration of 2 mg mL^−1^ to 0.5 mg mL^−1^ in PBS. The test antibody solution was prepared by diluting MaF antibodies from 4.9 mg mL^−1^ to 0.5 mg mL^−1^ in PBS (pH 7.4). The running buffer consisted of 0.05% (w%) Tween 20 and 2% sodium caseinate in PBS, and antigen solutions were prepared in two variants: one containing 200 ng mL^−1^ human ferritin antigen and a negative control without the antigen. For conjugate application, 22 μL of the conjugate solution was applied onto fiberglass pads, which were then dried in an incubator at 37 °C overnight. Test and control lines were applied on 3 mm × 25 mm nitrocellulose membranes using an automatic Nano-Plotter dispenser (Gesim, Radeberg, Germany) with dedicated software, applying two rows per antibody line, with an X-axis spacing of 0.45 mm, Y-axis spacing of 0.40 mm, and a drop volume of 4800 pL, positioning the test line at 10.5 cm and the control line at 16.5 cm from the base of the membrane. The membranes were dried at 37 °C overnight, after which all LFA components were assembled with 1 mm overlap. Subsequently, 100 μL of the running buffer, either containing ferritin or blank, was applied to each assembled test, performed in duplicate. After approximately 5 min, a substrate solution containing 1 mM NaBH_4_, 2 mM MTT, and 0.01 mM DTPA in PBS was applied directly to the membrane, and the results were evaluated visually approximately 5 min later. For comparison, another LFA for ferritin detection was developed using the same principle but employed N,N-diethyl-p-phenylenediamine sulfate (DEPDA) and 4-hydroxy-1-naphthalenesulfonic acid sodium salt (NSA) as chromogenic substrates, with H_2_O_2_ as the oxidizing agent, according to the method described by Mulvaney et al. [42]. Both assays were then analyzed using ImageJ (version v1.54g), which converted the images to 8-bit grayscale and quantified the grayscale intensities of the test line and background signal [43].

## 3. Results and Discussion

### 3.1. Nanoparticle Characterization

**Figure 1:**
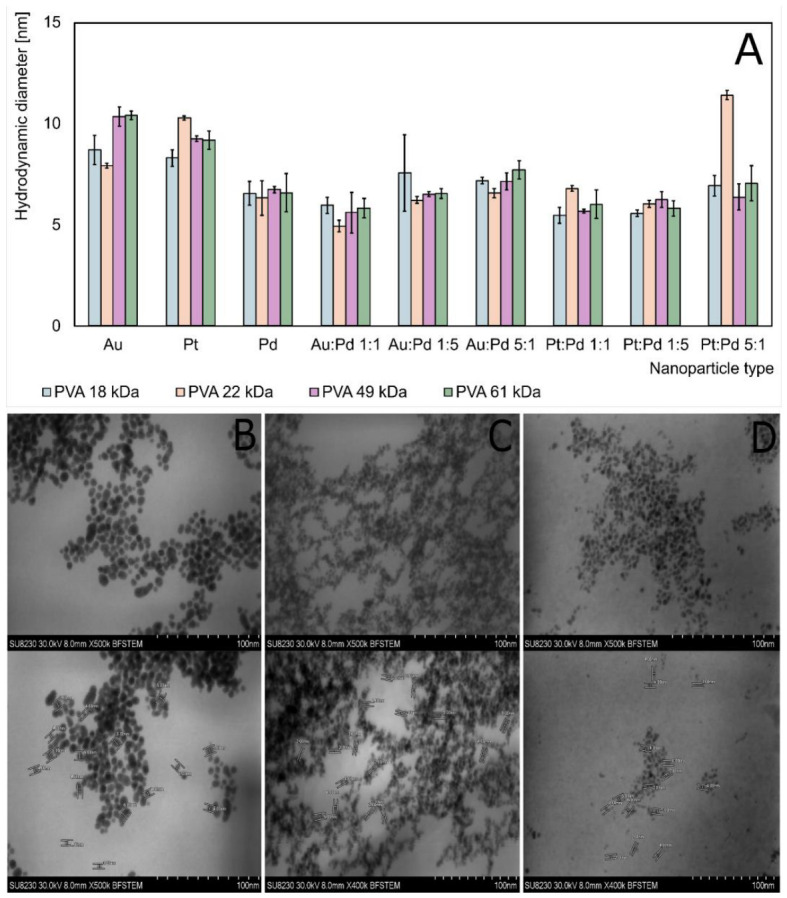
Size and morphology characterization of nanoparticles: (**A**)—hydrodynamic diameter DLS measurement of mono- and bimetallic (metals combined in molar ratio of 1:1, 1:5 and 5:1) nanoparticles stabilized with poly(vinyl alcohol) of various average molecular mass: 18 kDa, 22 kDa, 49 kDa and 61 kDa; (**B**)—bright field STEM image of Au nanoparticles stabilized with PVA 49 kDa; (**C**)—bright field STEM image of Pt nanoparticles; (**D**)—bright field STEM image of Au:Pd 1:1 nanoparticles.

The synthesis of each nanoparticle type under the selected conditions was successful, as confirmed by visual observation, dynamic light scattering (DLS), and scanning transmission electron microscopy (STEM) and Energy-Dispersive X-ray spectroscopy (EDX) analyses. Upon the addition of sodium borohydride (NaBH_4_), the reaction mixture changed color, and no visible agglomerates were observed after the synthesis was completed. Size determination using both spectroscopic and microscopic techniques further confirmed the successful formation of mono- and bimetallic nanoparticles. As shown in Figure 1A, the hydrodynamic diameter of the synthesized nanoparticles ranged from 5 to 12 nm. The exact values were presented in Table S1.

To validate the DLS results, STEM imaging was performed, and the obtained data revealed spherical nanoparticles with diameters consistent with those determined by DLS, which is demonstrated in Figure 1B–D. It is important to note that DLS measures the hydrodynamic diameter, which includes both the nanoparticle and the surrounding solvent layer. For example, the DLS results for palladium nanoparticles (PVA, 49 kDa) showed a hydrodynamic diameter of 6.75 ± 0.17 nm, while STEM measurements indicated a core diameter of approximately 4 nm. This distinction explains the slight differences observed between the DLS and STEM size measurements. The two techniques are complementary, as DLS provides information based on a large number of particles, offering statistically representative size distribution, while STEM offers detailed insight into nanoparticle shape and core size (without the solvent layer), but from a much smaller sample population. EDX analysis, presented in Figure S1, confirmed a uniform elemental distribution of Pd, Au, and Pt, indicating the formation of bimetallic nanostructures rather than a mixture of two distinct monometallic nanoparticles. These findings were in agreement with those reported by Bianchi et al., who described bimetallic nanoparticles comprising Au, Pt, and Pd prepared by rapid chemical reduction using NaBH_4_ as the reducing agent [44]. Moreover, spectroscopic measurements shown in Figure S2 showed that monometallic gold nanoparticles exhibited a characteristic plasmon resonance peak around 520 nm, whereas this peak was absent for bimetallic gold–palladium nanoparticles, even at a high gold content (5:1 molar ratio). This observation further supports the formation of bimetallic structures, as the presence of separate monometallic gold nanoparticles would be expected to produce a distinct gold plasmon peak in the spectrum. Synthesis using a high concentration of a strong reducing agent (NaBH_4_) in the presence of poly(vinyl alcohol) (PVA) proved to be an effective route for obtaining nanoparticles characterized by good stability and uniform nanoscale size distribution. The evaluation of PVA stabilizers with different molecular weights and degrees of hydrolysis demonstrated that each variant could efficiently stabilize the nanostructures while yielding nanoparticles of small size.

### 3.2. Investigation of the Catalytic Reduction of MTT by Nanoparticles

To assess the catalytic activity of the synthesized nanoparticles, the reduction of 4-NP was employed as a model reaction, enabling comparison with previously reported catalytic systems. However, a direct comparison of catalytic activity across reported studies can be challenging, since reaction conditions such as the choice and concentration of the reducing agent, the solution pH, or the concentration of nanoparticles play a significant role in determining reaction kinetics. Moreover, the Michaelis–Menten model, commonly used to describe enzyme kinetics, may not be directly applicable to nanozymes. Unlike natural enzymes, nanozymes typically lack strict substrate specificity and may catalyze multiple parallel reactions, including the generation of reactive oxygen species or hydroxyl radicals. As a result, the assumption of a well-defined and constant enzyme–substrate complex, fundamental to the Michaelis–Menten framework, is not met in these nanozyme systems. For example, Panferov et al. [45] compared 14 different nanoparticles, demonstrating up to a fourfold variation in the reported catalytic activity of the same nanozyme when evaluated under different experimental conditions. Additionally, nanozyme-based catalysis is generally considered a heterogeneous process that occurs on the nanoparticle surface, whereas enzyme catalysis typically operates via a homogeneous mechanism. This fundamental difference further limits the applicability of conventional kinetic models. For this reason, gold nanoparticles, widely recognized as efficient nanozymes for 4-NP reduction, were selected as a reference for evaluating the reduction efficiency of the synthesized nanoparticles. A schematic representation of the proposed mechanism for the nanozyme-catalyzed reduction of 4-NP is presented in Equation (1) [46].



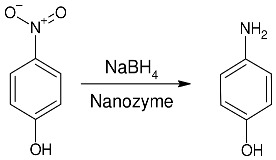

(1)


Equation (1). Schematic representation of the nanozyme-catalyzed reduction of 4-nitrophenol using sodium borohydride as the reducing agent.

The catalytic activities were compared under identical conditions for each reaction set. To ensure that the nanoparticles acted as catalysts in the proposed reactions, control experiments using the substrate with nanoparticles and the substrate with the reducing agent were also performed. A schematic representation of the proposed mechanism for the nanozyme-catalyzed reduction of MTT is presented in Equation (2). The presented approach enables direct comparison of nanozyme activity without requiring analysis of detailed reaction mechanisms, such as reaction orders or catalytic pathways, which have not yet been established for the reduction of tetrazole salts in the presence of nanoparticles.



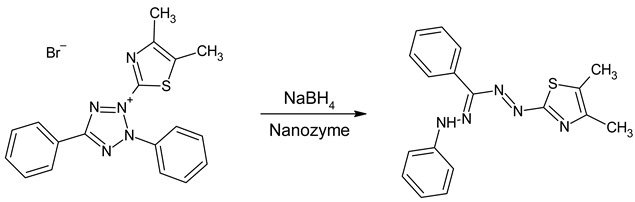

(2)


Equation (2). Schematic representation of the nanozyme-catalyzed reduction of MTT salt using sodium borohydride as the reducing agent.

The rate of absorbance change was determined by fitting a linear regression to the experimentally measured absorbance values over time. To enable quantitative comparison of catalytic activity among different nanozymes, apparent zero-order kinetics were assumed within the linear reaction range. The apparent reaction rate constant, k, was calculated from the slope of each regression line using Equation (3). An example of the kinetic curve captured for each measurement is shown in Figure S3.

(3) y=kx+b

Equation (3). Absorbance change rate equation: k—absorbance change over time; y—absorbance; x—time; b—constant.

The minimum number of points for linear regression was set to 7 consecutive measurements in the linear range. Each measurement was performed with at least 5 repetitions. The measurements were recorded with 15-s intervals for approximately 10 min each. The coefficient of determination for each linear fit was required to be greater than 0.90. It is important to note that if these standards were not met, the experiment was adjusted with a different nanoparticle dilution to obtain a good linear fit. In this study, the relative catalytic activity of the synthesized nanozymes was assessed by the rate of change in absorbance over time (increase for MTT or decrease for 4-NP). Because the nanoparticles differed in elemental composition, bimetallic structure, and the molecular weight of the stabilizing polymers, comparison based on the mass of dried material could be misleading. The molecular weight of the polymer influences the total sample mass but does not directly affect the catalytic centers responsible for the reaction. Therefore, a kinetic parameter was introduced and defined as the absorbance change rate normalized to the molar concentration of the metal ions forming the nanoparticles present in the catalytic assay ($\Delta A\;h^{-1}\;µM_{Me}^{-1}$), which was calculated based on Equation (4).

(4) $$Absorbance\;change\;rate=\frac{k}{C_{mol}}$$

Equation (4). Calculation of the relative kinetic performance parameter for nanozymes comparison; k—absorbance change over time; C_mol_—molar concentration of metal content in the nanoparticles solution.

**Figure 2:**
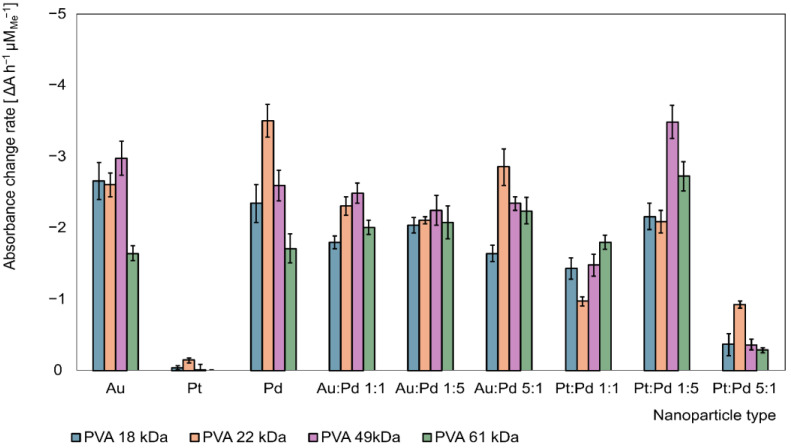
Comparison of the absorbance change rate of nanoparticles catalyzing the reduction of 4-nitrophenol, indicating their catalytic activity, based on the absorbance decrease rate over a set amount of time (from yellow 4-NP to clear 4-aminophenol). Measurement of absorbance in a fixed wavelength mode (400 nm).

This approach enabled a more meaningful comparison of catalytic activity among the synthesized nanozymes and with results reported in other studies. This simplification is justified by the early-stage nature of research on tetrazolium salt reduction for diagnostic applications, as, to the best of our knowledge, this is the first demonstration of tetrazolium salt reduction catalyzed by nanoparticles. A more comprehensive mechanistic investigation should be considered in future studies. Both gold and palladium nanoparticles, as well as their bimetallic counterparts, exhibited high catalytic activity toward the reduction of 4-NP. The obtained results are shown in Figure 2, and the detailed values for each nanoparticle type are summarized in Table S2.

The highest rate of absorbance decrease (yellow to colorless) was observed for Pd_mono_ and Pt:Pd (1:5 molar ratio) nanoparticles, both exhibiting values of −3.5 $\Delta A\;h^{-1}\;µM_{Me}^{-1}$. In contrast, platinum-based nanoparticles exhibited the lowest overall catalytic activity. The absorbance change observed for Pt_mono_ nanozymes stabilized by PVA 22 kDa reached −0.1 $\Delta A\;h^{-1}\;µM_{Me}^{-1}$; the exception was the Pt:Pd bimetallic nanoparticles at a 1:5 molar ratio, as already stated. This is likely due to the higher palladium content in their composition, which significantly contributes to overall catalytic efficiency. Control experiments were also conducted without the addition of nanoparticles or sodium borohydride, both showing no significant changes in absorbance. This confirms that the synthesized nanoparticles served as the catalytic agents responsible for the reduction of 4-nitrophenol by NaBH_4_.

**Figure 3:**
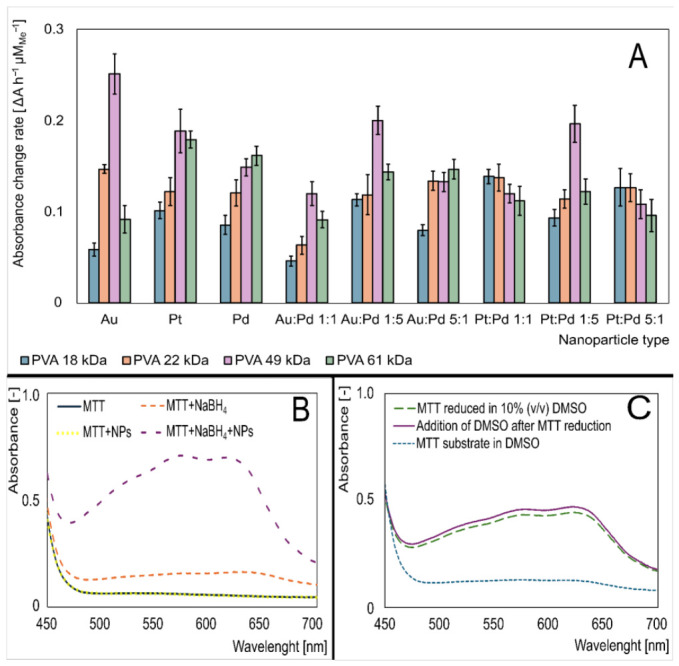
Evaluation of catalytic activity of nanozymes towards MTT salt: (**A**)—Comparison of absorbance change rate of nanoparticles catalyzing the reduction of 2-(4,5-Dimethylthiazol-2-yl)-3,5-diphenyl-2H-tetrazol-3-ium bromide, based on absorbance increase rate over a set amount of time (from yellow MTT to violet-blue formazan). Measurement of absorbance in a fixed wavelength mode (570 nm). (**B**)—Absorption spectra of MTT in 10% (*v*/*v*) DMSO in universal buffer (pH 9.0) recorded for the MTT substrate, MTT with added nanoparticles, MTT with added NaBH_4_, and MTT with both nanoparticles and NaBH_4_. (**C**)—Absorption spectrum of MTT reduced in the presence of nanoparticles and NaBH_4_ in universal buffer containing DMSO, as well as in universal buffer, followed by the addition of DMSO after the reaction occurred.

Building upon the demonstrated ability of the synthesized nanozymes to catalyze the reduction of 4-nitrophenol with sodium borohydride, the potential of tetrazolium salts as substrates for nanozyme-amplified PoC systems was further explored. The catalytic activity of the synthesized nanoparticles was compared with the reduction of MTT tetrazole salt by sodium borohydride. As shown in Figure 3A, an increase in the absorbance change rate was observed for gold, palladium, and platinum nanoparticles, as well as their bimetallic counterparts, in contrast to the reduction of 4-nitrophenol, in the case of which platinum displayed limited catalytic activity.

The highest absorbance change rates were obtained for Au_mono_, Pt_mono_, and Au:Pd (1:5 molar ratio) nanoparticles, showing catalytic activity of 0.25, 0.19, and 0.20 $\Delta A\;h^{-1}µM_{Me}^{-1}$, respectively. Comparison of stabilizing agents used during synthesis revealed that PVA with the highest molecular weight (M_w_ = 49 kDa) led to nanozymes with superior catalytic activity (0.20 $\Delta Ah^{-1}µM_{Me}^{-1}\;$ for Pt:Pd, 1:5 molar ratio) compared to other PVA variants (0.09 $\Delta A\;h^{-1}µM_{Me}^{-1}\;$ for PVA 18 kDa, 0.11 $\Delta A\;h^{-1}µM_{Me}^{-1}\;$ for PVA 22 kDa, and 0.12 $\Delta A\;h^{-1}µM_{Me}^{-1}\;$ for PVA 61 kDa for respective nanoparticle compositions). The numerical values for each nanoparticle type were given in Table S3. Since each synthesis mixture contained the same polymer amount (wt%), this likely led to fewer, longer PVA chains adsorbing on the nanozyme surface, thereby increasing the number of accessible active sites while maintaining sufficient nanoparticle stability. The nanoparticles stabilized with the longest PVA polymers (61 kDa) exhibited lower catalytic activity than those stabilized with 49 kDa PVA, likely due to stronger, more extensive interactions of the longer chains with the nanoparticle surface, resulting in a thicker polymeric shell that limited access to catalytic sites. In the tetrazolium salt reduction assay, control experiments with the substrate alone, with nanoparticles, or with a reducing agent were also performed. The addition of nanoparticles alone did not induce any change in MTT absorbance. In contrast, NaBH_4_ caused a slight reduction of MTT even in the absence of nanoparticles. However, this effect was considerably smaller than when the same amount of NaBH_4_ was combined with nanoparticles (Au:Pd 1:1, PVA 49 kDa), as shown in the absorption spectrum in Figure 3B. It is important to note that DMSO is an essential component of the substrate solution, as reduced formazan is not soluble in water and forms crystalline structures upon reduction of soluble MTT. The insolubility of formazan is advantageous for PoC applications, but it can lead to unreliable spectrophotometric measurements due to aggregation of the solid product, which may yield inaccurate readings. To ensure that DMSO does not influence MTT reduction in the presence of nanoparticles (Au:Pd 1:1, PVA 49 kDa), experiments were conducted in two ways: with DMSO present during the reduction and with DMSO added after a set reaction time of 10 min. Both solutions were then examined spectrophotometrically, and the resulting absorbance spectra were compared, as shown in Figure 3C. No significant differences were observed between the two approaches, indicating that DMSO does not substantially interfere with MTT reduction. Moreover, the effect of DMSO concentration in the substrate solution was investigated over the range of 2–20% (*v*/*v*). As shown in Figure S5, the addition of 10% DMSO provided the most reproducible results, yielding the lowest variability among replicates. The relative standard deviation was approximately 2%, whereas DMSO concentrations of 2%, 5%, 15%, and 20% resulted in deviations exceeding 5% from the mean value.

**Figure 4:**
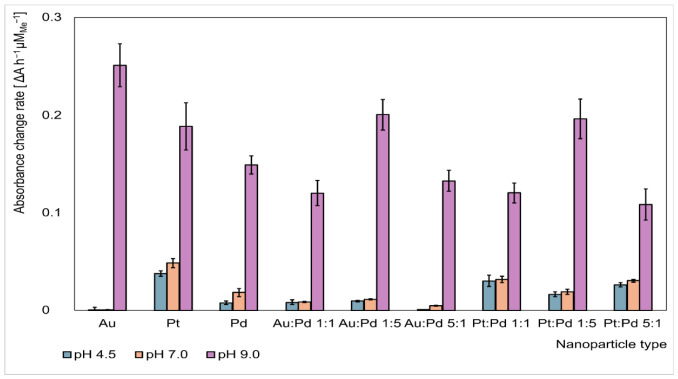
Comparison of the absorbance change rate of nanoparticles catalyzing the reduction of MTT salt utilizing NaBH_4_ as reducing agent in acidic, neutral, and basic environments using Britton–Robinson universal buffer set to pH: 4.5, 7.0, and 9.0.

Since the pH of the reaction medium plays a crucial role in determining catalytic performance, the reactions were evaluated under acidic, neutral, and basic conditions, with particular emphasis on NaBH_4_ as the reducing agent. Grzeschik et al. reported a clear pH dependence of catalytic activity toward 4-NP reduction, proposing two distinct pathways: a slower hydride-reduction mechanism and a faster hydrogen-reduction pathway arising from borohydride hydrolysis, which is slowed under basic conditions, thereby providing a more stable reaction environment for direct comparison of reduction efficiency [47]. As shown in Figure 4, the relative rate of absorbance changes for MTT reduction in pH 4.5, 7.0, and 9.0 resulted in values of 0.04, 0.05, and 0.19 $\Delta A\;h^{-1}µM_{Me}^{-1}$, respectively, for the platinum monometallic nanoparticles. For both monometallic and bimetallic nanoparticles, the highest catalytic activity was consistently observed at pH 9.0. Gold nanoparticles exhibited the strongest pH dependence, showing negligible catalytic activity at pH 4.5 and 7.0, whereas at pH 9.0 they displayed the highest activity among all tested nanozymes, reaching 0.25 $\Delta A\;h^{-1}µM_{Me}^{-1}$. A similar trend was observed for gold-containing bimetallic nanoparticles. For example, Au nanoparticles with a 1:1 molar ratio exhibited catalytic activities of approximately 0.01 $\Delta A\;h^{-1}µM_{Me}^{-1}$ at both pH 4.5 and 7.0, while their activity increased twelvefold at pH 9.0, reaching 0.12 $\Delta A\;h^{-1}µM_{Me}^{-1}$. For this reason, Au:Pd nanoparticles with a 1:1 molar ratio were further investigated over the pH range of 8.0–10.0. The results are presented in Figure S4. At pH values below 9.0, the measurements exhibited relatively high standard deviations, likely due to ongoing borohydride hydrolysis and the associated formation of hydrogen bubbles that interfered with absorbance measurements. As the pH increased, the catalytic activity also increased, reaching normalized catalytic activity values of 0.08, 0.09, and 0.09 $\Delta A\;h^{-1}µM_{Me}^{-1}$ at pH 9.0, 9.5, and 10.0, respectively. These results suggest that the catalytic activity approaches a plateau above pH 9.0, with no substantial improvement observed at higher pH values. It is important to note that the observed changes in catalytic activity may be influenced by differences in nanoparticle stability at different pH values. Therefore, the ζ-potential of Au:Pd nanoparticles with a 1:1 molar ratio was measured at pH 4.5, 7.0, and 9.0, as well as in deionized water. The results are summarized in Table S4. Although the ζ-potential decreased from −38.9 mV at pH 9.0 to −27.9 mV at pH 4.5, all measured values remained below −20 mV, a threshold commonly associated with colloidally stable nanoparticle suspensions. Furthermore, nanoparticle stability in these systems is not governed solely by electrostatic repulsion. The adsorbed polymer layer can also provide steric stabilization, further reducing the likelihood of aggregation even under conditions of reduced surface charge. Therefore, the observed differences in catalytic activity are unlikely to result from nanoparticle destabilization or aggregation and are more likely attributable to the effect of pH on the catalytic process itself.

Consistent with literature findings, the highest catalytic activity—over four times greater than under acidic conditions—was also observed in the presented system in a basic environment. Moreover, the color change from yellow MTT to violet-blue formazan was confirmed by naked-eye observation. As previously mentioned, the pH of solutions used in point-of-care devices is usually close to 7.0 and also commonly covers more basic conditions, supporting the applicability of tetrazole salts in point-of-care settings.

### 3.3. Design and Evaluation of a Proof-of-Concept Lateral Flow Assay Employing MTT for Signal Generation

**Figure 5:**
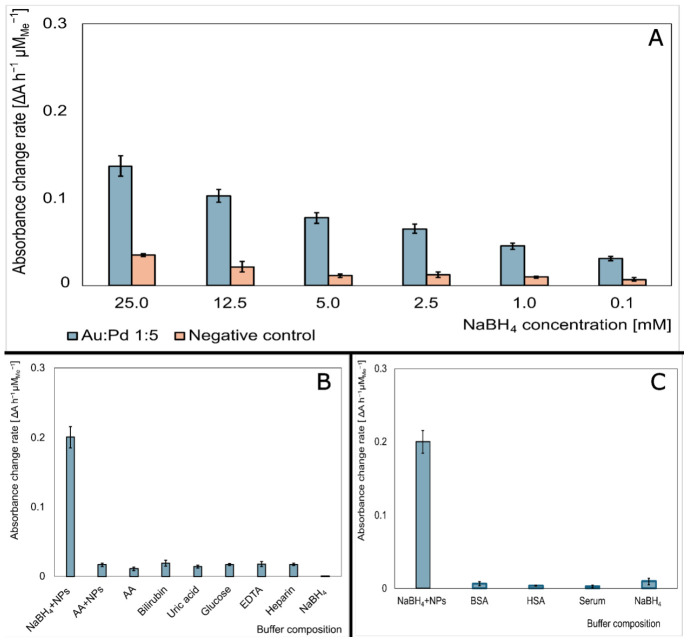
Absorbance change rate comparison between various interferent additions and substrate solution composition for tetrazole salt applicability in PoC devices: (**A**)—selection of reducing agent (NaBH_4_) working concentration for PoC application; (**B**)—study on ascorbic acid (AA) interference on non-intended reduction of MTT salt with and without the presence of nanoparticles (NPs); (**C**)—study on protein and human serum interference on non-intended reduction of MTT salt.

To further support the goal of employing tetrazolium salts as components (substrates) for a signal-generation enhancement approach for diagnostic applications, the influence of NaBH_4_ concentration on both analytical signal and background noise was also examined. The signal-to-noise ratio is a critical parameter for any analytical system—particularly for PoC diagnostic devices, which are intended for use by untrained individuals who can misinterpret results [48]. Therefore, it is essential to establish experimental conditions that provide a distinct, measurable signal while minimizing background signal generation in the absence of nanoparticles. As shown in Figure 5A, application of NaBH_4_ at a concentration of 25.0 mM led to the highest absorbance change rate of 0.14 $\Delta A\;h^{-1}µM_{Me}^{-1}$. However, a considerable increase in absorbance, equal to 0.03 $\Delta A\;h^{-1}µM_{Me}^{-1}$, was also observed without the addition of nanoparticles, making this concentration less suitable for use in various biosensing formats.

For that reason, 1.0 mM concentration was chosen for further studies, since it provided a sufficient rate of absorbance increase of 0.05 ± 0.004 $\Delta A\;h^{-1}µM_{Me}^{-1}$, resulting in a clear appearance of colored product; moreover, there was no visible color development in the samples with no nanozymes added. A relatively low chemical concentration is an additional advantage that will contribute to lower manufacturing costs in the future. The greater change in absorbance observed at higher sodium borohydride concentrations can be attributed to the dual-reduction mechanism of NaBH_4_, as discussed above. As the borohydride concentration increases, the hydrogen reduction pathway becomes more dominant relative to the fixed concentration of MTT, leading to non-catalytic substrate reduction. Additionally, impurities in the reagents or sample matrix may contribute to tetrazolium salt reduction, as trace metal cations can act as unintended catalysts. Such impurities, even at trace levels, can accelerate redox reactions or initiate secondary reaction pathways, leading to increased background signal and reduced assay precision when an excess of reducing agent is present, which is especially critical in colorimetric systems intended for diagnostic applications.

To assess the suitability of tetrazolium salt reduction for use in PoC systems, several factors that could potentially cause unintended substrate reduction in biological samples were also examined. Chakrabarti et al. reported elevated nonspecific MTT reduction in one of the cell culture media they tested, identifying ascorbic acid (AA) as the primary reducing agent, with retinol acting as a catalyst [49]. Since their study focused on concentrations relevant to cell culture conditions, the impact of ascorbic acid at levels representative of human blood and serum was investigated, as these matrices are critical in medical diagnostics. The physiological concentration of AA in adult blood typically ranges from 0.6 to 2.0 mg L^−1^ [50]. As shown in Figure 5B, no significant increase in absorbance change rate was observed even at the upper physiological limit of 20.0 mg L^−1^ AA. Several potential interferents commonly present in human blood and known to exhibit antioxidant properties, including bilirubin, uric acid, and glucose, were evaluated at their physiological concentrations of 12.5 µg mL^−1^, 60 µg mL^−1^, and 1.0 mg mL^−1^, respectively [51,52,53]. In addition, EDTA and heparin, which are widely used as anticoagulants in blood collection tubes, were investigated as potential interferents at concentrations representative of those used in commercial blood collection systems, namely 2.2 mg mL^−1^ for EDTA and 0.14 mg mL^−1^ for heparin [54,55]. Although all tested interferents increased the rate of absorbance change compared with the borohydride-only control, none produced a substantial enhancement in catalytic activity, as shown in Figure 5B. These findings suggest that the MTT-based system is suitable for use in complex biological matrices, a hypothesis that was further evaluated using serum samples. Moreover, in practical applications, biological samples are typically diluted before analysis. For serum-based assays, sample concentrations of 10% (*v*/*v*) or lower are commonly employed, further reducing the concentrations of potential interferents and minimizing their impact on assay performance. Another key factor to consider was the reducing activity of human serum albumin (HSA), the most abundant protein in blood plasma. Funk et al. reported an increase in absorbance in cell culture media containing HSA. In their study, various proteins were tested for potential interference in the MTT cell viability assay. Moreover, the authors concluded that cysteine residues (containing thiol groups) in albumin were responsible for the enhanced reduction of tetrazolium salts [56]. As with ascorbic acid, the observed effect was studied primarily under conditions suitable for cell culture. The physiological concentration of albumin in human serum ranges from 35 to 50 mg mL^−1^ [57]. As shown in Figure 5C, no significant interference in MTT reduction was observed even at the upper physiological limit of HSA or the corresponding BSA concentration. Compared with cell viability assays, PoC systems require lower component concentrations, shorter incubation times, lower operating temperatures, and additional sample dilution, thereby minimizing the risk of unintended substrate reduction and background signal generation. To assess the influence of additional blood and serum components not previously discussed and to account for possible synergistic effects within such complex matrices, the impact of commercially available human serum on unintended MTT reduction was further examined. As shown in Figure 5C, a human serum sample diluted 1:10 in buffer did not cause any noticeable increase in the rate of absorbance change compared to the negative control. In contrast, samples containing nanozymes exhibited a significantly greater change in absorbance, confirming that the observed signal arises from the nanoparticles’ catalytic activity.

**Figure 6:**
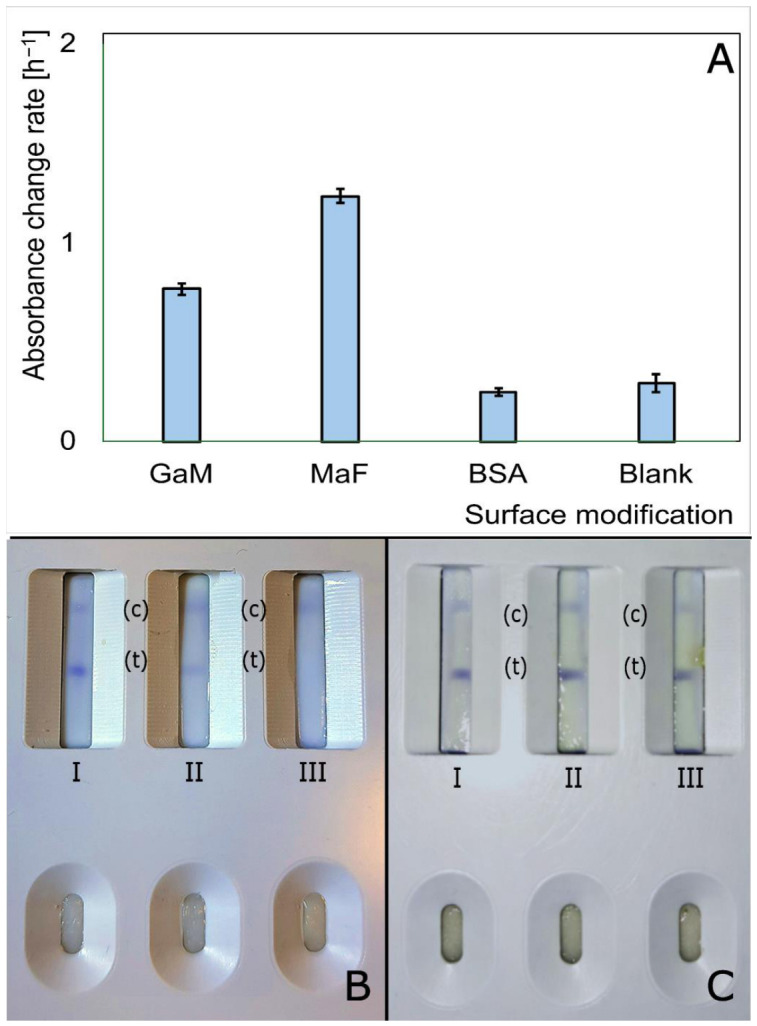
Absorbance change rate comparison as a result of the immunochemical reaction between nanozyme-antibody conjugate and surface antibody. Proof-of-concept lateral flow assay preparation: (**A**)—conjugation efficiency evaluation of mouse anti-human ferritin antibodies to Au:Pd bimetallic nanoparticles (1:5 molar ratio, PVA 49 kDa) by direct capture of conjugate by goat anti-mouse (GaM) antibodies and sandwich type interaction utilizing complementary, monoclonal mouse anti-human ferritin (MaF) antibody and human liver ferritin antigen with negative control for unspecific conjugate interaction with bovine serum albumin (BSA); (**B**)—image of lateral flow assay consisting of GaM control lines (c) and complementary, monoclonal MaF test lines (t), the LFAs were carried out with: I, II—ferritin antigen spiked running buffer (200 ng ml^−1^); III—running buffer; (**C**)—image of a second batch of lateral flow assays consisting of GaM (c) and MaF test lines (t), the LFAs I, II and III were carried out with running buffer spiked with ferritin antigen (200 ng ml^−1^).

To demonstrate the applicability of tetrazolium salt reduction reactions for signal generation in PoC settings, a lateral flow assay for ferritin detection was developed as a proof-of-concept using MTT as the substrate. It is important to note that this is only a feasibility demonstration of the proposed setting, based on well-established procedures that use peroxidase substrates to generate signals. Nanozyme conjugation was performed via passive adsorption, followed by a nanozyme-linked immunosorbent assay (NLISA) to verify antibody attachment to the nanoparticle surface. Figure 6A presents the results for both direct capture and sandwich-type antibody interactions, confirming that mouse anti-human ferritin antibodies successfully formed stable nanozyme-antibody conjugates with Au:Pd nanoparticles (1:5 molar ratio, PVA 49 kDa).

Negative control exhibited a relatively high signal of 0.3 h^−1^, equal to 32.2% of the signal achieved for GaM. Nevertheless, the results for the actual samples were much higher (1.2 h^−1^), which proved the conjugation procedure was successful. Figure 6B,C illustrate a nanozyme-enhanced lateral flow assay with mouse anti-ferritin antibodies forming the test line, goat anti-mouse antibodies as the control line, and antibody-nanozyme conjugates as the signal generation component. The assay was performed twice, two weeks apart, using a running buffer with 200 mg mL^−1^ ferritin antigen and a buffer without antigen as a negative control. Upon addition of MTT substrate solution, a distinct colorimetric signal developed in the designated test area (t) for the spiked sample (Figure 6(BI,BII)), while no test line was observed when only the buffer without antigen was applied (Figure 6(BIII)). The colored lines became visually distinguishable within approximately 5 min of substrate addition. Moreover, the LFAs prepared in a second independent run (Figure 6(CIIII)) produced comparable results when using the same protocol, confirming the reproducibility of the signal-generation concept based on tetrazole salt reduction. Furthermore, the reduced formazan product remained in the designated areas due to its precipitation upon MTT reduction; no unintentional flow of the product was observed. The control line (c) was developed for positive and negative control experiments to verify proper sample flow. In addition, the low intensity of the noise signal was observed and could be further reduced through assay optimization in future studies. The oxidation-based LFAs are shown in Figure S6. Four repetitions of positive samples containing 200 ng mL^−1^ of ferritin were tested in each case, while no unspecific analytical signal developed on the test line of the negative sample (first strip test); however, the background signal remained high. Table 1 summarizes the grayscale analysis results and compares the signal generated by reduction- and oxidation-based substrates.

The oxidation-based assay using the DEPDA + NSA substrate showed a mean difference of 12 units between the test line and background signal. In contrast, the reduction-based MTT assay produced a 47-unit difference, approximately four times higher, indicating significantly improved signal discrimination. These results suggest that the MTT-based system provides a higher analytical contrast, which may translate into improved sensitivity and more reliable signal interpretation in LFA-based detection.

## 4. Summary and Conclusions

In this work, the applicability of tetrazole salts as substrates in point-of-care diagnostics was investigated for the first time. Mono- and bimetallic gold, platinum, and palladium nanoparticles were synthesized using poly(vinyl alcohol) of various molecular weights as stabilizing agents and sodium borohydride as reducing agent. The chosen synthesis conditions yielded stable, well-dispersed, and uniform nanostructures, as confirmed by Dynamic Light Scattering, UV–Vis Spectroscopy, Scanning Transmission Electron Microscopy, and Energy-Dispersive X-ray Spectroscopy. The influence of PVA with different molecular weights and degrees of hydrolysis was also examined, revealing that all tested variants effectively stabilized the nanoparticles. Notably, PVA with a higher molecular weight (49 kDa) yielded nanozymes with superior catalytic activity, attributed to fewer polymer chains adsorbed per nanoparticle and greater accessibility of the active catalytic sites. The reduction of 4-nitrophenol, a model substrate, as a probe of catalytic activity towards substrate reduction using sodium borohydride was further investigated. Gold and palladium nanoparticles, as well as their bimetallic counterparts, exhibited high catalytic activity, whereas platinum-based nanoparticles showed lower activity, except when combined with palladium at a 1:5 molar ratio. These findings align with the known catalytic efficiency of palladium and gold systems for 4-NP reduction. Control experiments confirmed that the observed reactions occurred exclusively in the presence of synthesized nanoparticles, demonstrating their role as active catalysts that mimic the activity of mitochondrial reductases, e.g., succinate dehydrogenase. Building upon these findings, the nanoparticles were evaluated as catalysts for the reduction of tetrazolium salts, with MTT selected as a model substrate given its relevance to colorimetric assays. The observed increase in absorbance during MTT reduction confirmed that the synthesized nanoparticles can catalyze reactions that generate measurable colorimetric signals, which are essential for point-of-care diagnostic applications. Further optimization of reaction parameters indicated that the buffer pH strongly influenced the catalytic activity. The highest reaction rates were observed under basic conditions (pH 9.0). Similarly, the investigation of the impact of NaBH_4_ concentration revealed that 1.0 mM yielded an acceptable ratio of the generated analytical signal to the background signal. The selected concentration ensured a clear colorimetric signal in the presence of nanoparticles and a negligible background signal in their absence, which is essential for PoC devices. To evaluate the selectivity of the proposed system under conditions relevant to biological samples, potential interferences from ascorbic acid, serum albumin, and commercial human serum were studied. The results showed no significant reduction of MTT even at the upper physiological limits of these compounds, demonstrating that the system remains selective and stable in complex biological environments. This finding is critical for ensuring reliability and minimizing false positive results in real diagnostic matrices. Finally, as a proof-of-concept demonstration, a nanozyme-based lateral flow assay for ferritin detection was developed, using MTT as a colorimetric substrate to enhance signal. The successful appearance of a visible signal consisting of precipitated reduced MTT (formazan) on the test line confirmed the nanozyme-catalyzed reduction of tetrazolium salts. In summary, this work establishes an effective synthetic route for producing catalytically active and stable mono- and bimetallic nanozymes. Their demonstrated ability to catalyze both 4-NP and tetrazolium salt reductions, combined with their compatibility with biological matrices, supports the potential of tetrazole salts for application in nanozyme-enhanced diagnostic devices. Although this study demonstrates the potential of MTT for point-of-care applications, there remains substantial room for further investigation, as it is the first time that nanomaterials have catalyzed the reduction of tetrazole salts. This research demonstrates that, in addition to oxidase-like nanoparticles, reductase-like nanozymes can also be employed for signal generation, expanding the toolkit for point-of-care diagnostic applications. Future research should focus on understanding the mechanism of tetrazolium reduction by nanozymes (especially CTC for simultaneous colorimetric and fluorescent readouts), identifying and evaluating substrates beyond tetrazole salts, and further optimizing and integrating these catalytic nanomaterials into novel PoC diagnostic platforms. The proposed approach can serve as an effective signal amplification strategy in PoC assays, offering a promising alternative to conventional peroxidase- or phosphatase-based amplification systems.

## Figures and Tables

**Table 1:** Grayscale intensity analysis of reduction-based LFA and oxidation-based LFA.

	MTT LFA			DEPDA + NSA LFA		
	Grayscale Intensity [−]	Mean [−]	Standard Deviation [−]	Grayscale Intensity [−]	Mean [−]	Standard Deviation [−]
Test Line	132	129	±7	175	189	±11
	134			189		
	135			191		
	117			202		
	127					
Background	189	176	±11	209	201	±6
	185			206		
	176			199		
	166			197		
	164			195		

## Data Availability

The data presented in this study are available on request from the corresponding author.

## Supplementary Materials

The following supporting information can be downloaded at: https://www.mdpi.com/article/10.3390/bios16070360/s1, Figure S1: STEM-EDX elemental analysis of nanoparticles suspension, including elemental dispersion maps and elemental composition graphs. (A) gold monometallic, PVA 49 kDa nanoparticles; (B) palladium monometallic, PVA 49 kDa nanoparticles; (C) gold-palladium bimetallic nanoparticles, PVA 49 kDa, 1:1 molar ratio; (D) gold-palladium bimetallic nanoparticles, PVA 49 kDa, 1:5 molar ratio; Figure S2: UV–Vis absorption spectra of the synthesized mono- and bimetallic gold- and palladium-based nanoparticle suspensions; Figure S3: Time-dependent change in absorbance visualizing the formation of formazan in an MTT assay, showing enhanced reduction in the presence of catalytic nanoparticles compared to the blank sample at 570 nm.; Figure S4: Comparison of pH-dependent catalytic activity of Au:Pd (1:5, 49 kDa) nanoparticles in MTT re-duction within the pH range of 8–10. Error bars represent the standard deviation of three inde-pendent measurements (n = 3); Figure S5: Effect of DMSO concentration (2–20%, *v*/*v*) on the catalytic activity of nanozymes. Au:Pd nano-particles (1:5, 49 kDa PVA) were used as a model catalytic system. Error bars represent the standard deviation of three independent measurements (n = 3); Figure S6: Oxidation-based lateral flow assay for ferritin detection employing DEPDA + NSA as chromogenic signal-generating substrates. Strip A: negative control; Strips B–E: positive control (200 ng mL−1 ferritin); Table S1: Hydrodynamic diameter of various nanoparticles based on DLS measurements; Table S2: Experimental data based on the absorbance decrease for 4-NP substrate reduction; Table S3: The values of the slope of linear functions fitted to the experimental data based on the absorbance increase for MTT substrate reduction; Table S4: pH-dependent ζ-potential measurements of Au:Pd (1:1) nanoparticles.
