# Supplementary material for: Exploring Tetrazolium Salt Reduction by Mono- and Bimetallic Nanoparticles as an Alternative Signal-Generation Strategy for Point-of-Care Diagnostics
Source: Biosensors (Basel). 2026 Jun 29;16(7):360. doi: 10.3390/bios16070360 (PMC13407067; doi:10.3390/bios16070360)
Supplement: Supplementary file 1 [file biosensors-16-00360-s001.zip › biosensors-4335628-supplementary.pdf]

biosensors

MDPI

Supporting Information

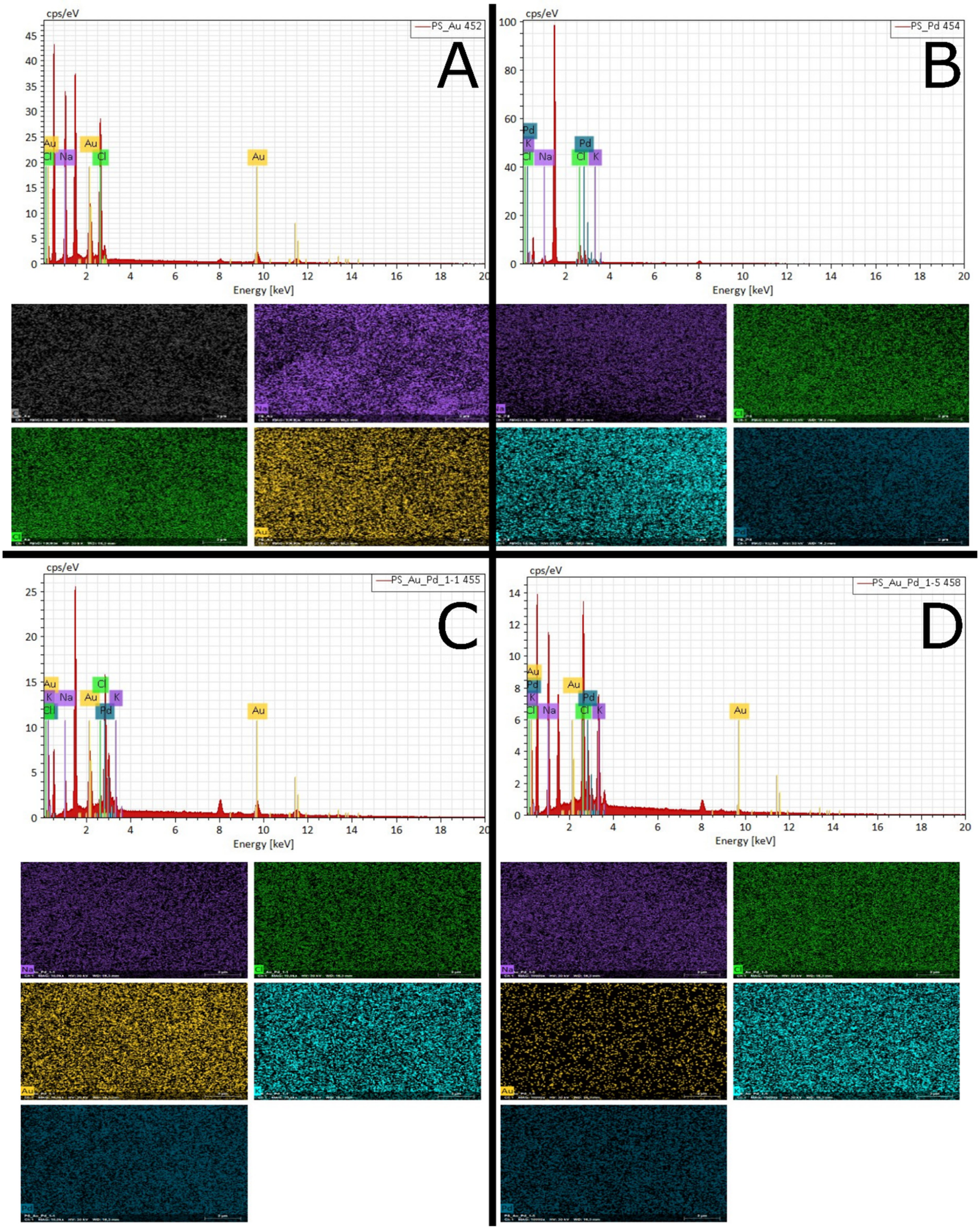

Figure S1. STEM-EDX elemental analysis of nanoparticles suspension, including elemental dispersion maps and elemental composition graphs. (**A**)gold monometallic, PVA 49 kDa nanoparticles; (**B**) palladium monometallic, PVA 49 kDa nanoparticles; (**C**) gold-palladium bimetallic nanoparticles, PVA 49 kDa, 1:1 molar ratio; (**D**) gold-palladium bimetallic nanoparticles, PVA 49 kDa, 1:5 molar ratio.

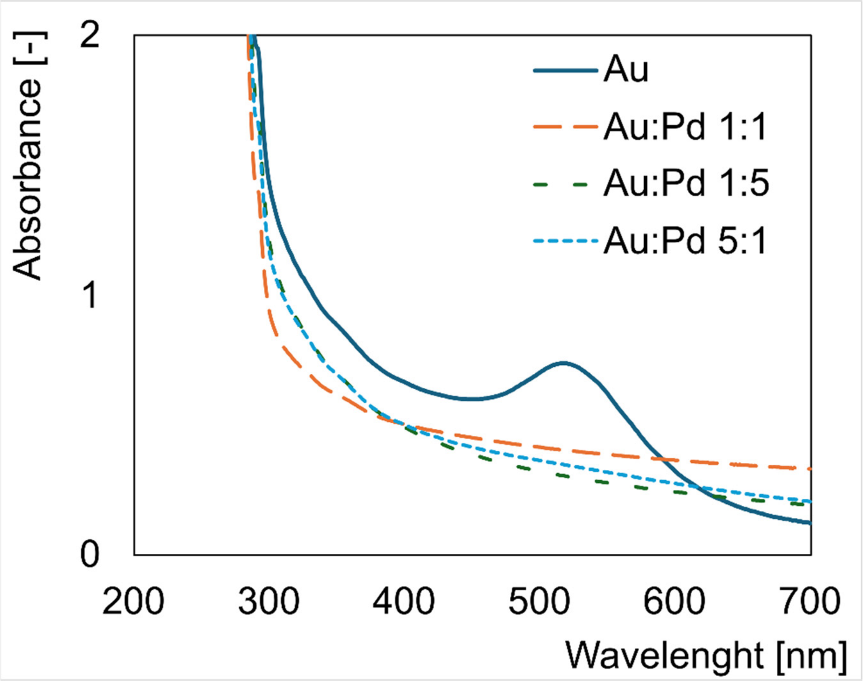

Figure S2. UV–Vis absorption spectra of the synthesized mono- and bimetallic gold- and palladium-based nanoparticle suspensions.

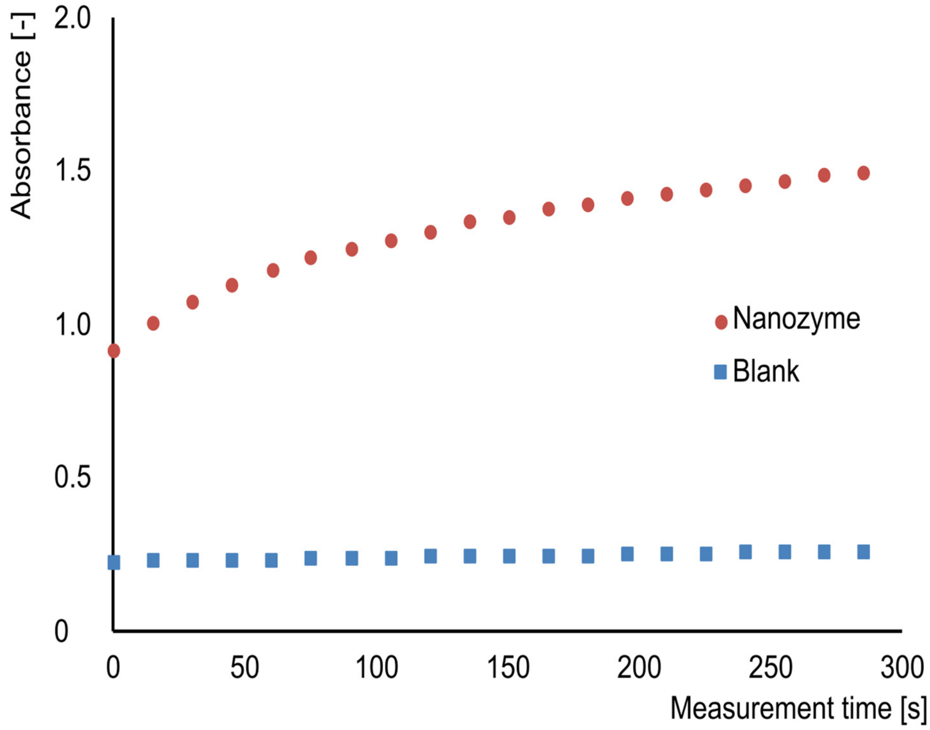

Figure S3. Time-dependent change in absorbance visualizing the formation of formazan in an MTT assay, showing enhanced reduction in the presence of catalytic nanoparticles compared to the blank sample at 570 nm.

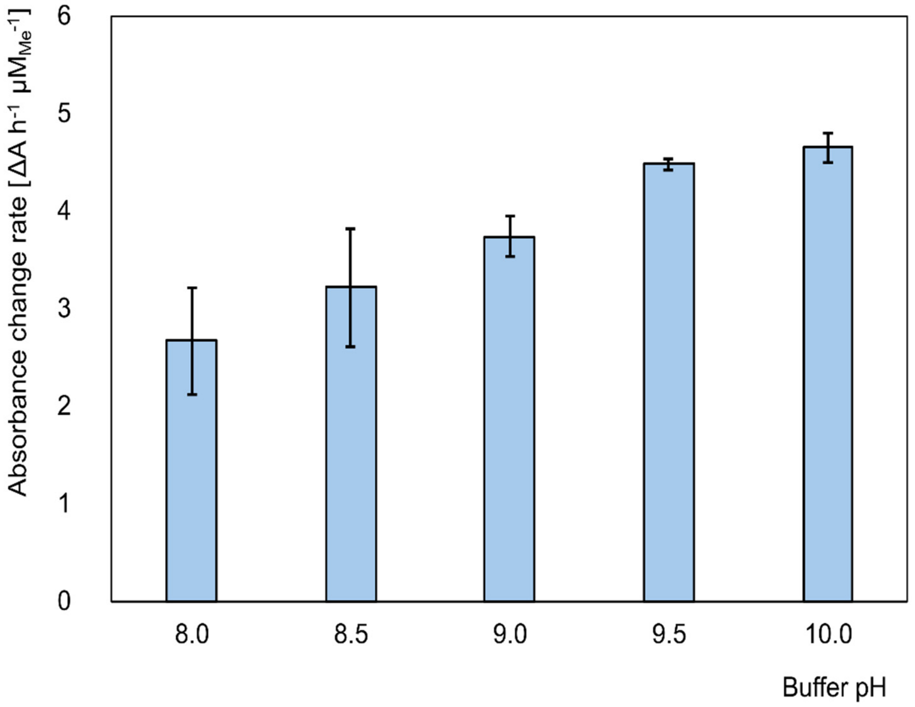

Figure S4. Comparison of pH-dependent catalytic activity of Au:Pd (1:5, 49kDa) nanoparticles in MTT reduction within the pH range of 8–10. Error bars represent the standard deviation of three independent measurements (n = 3).

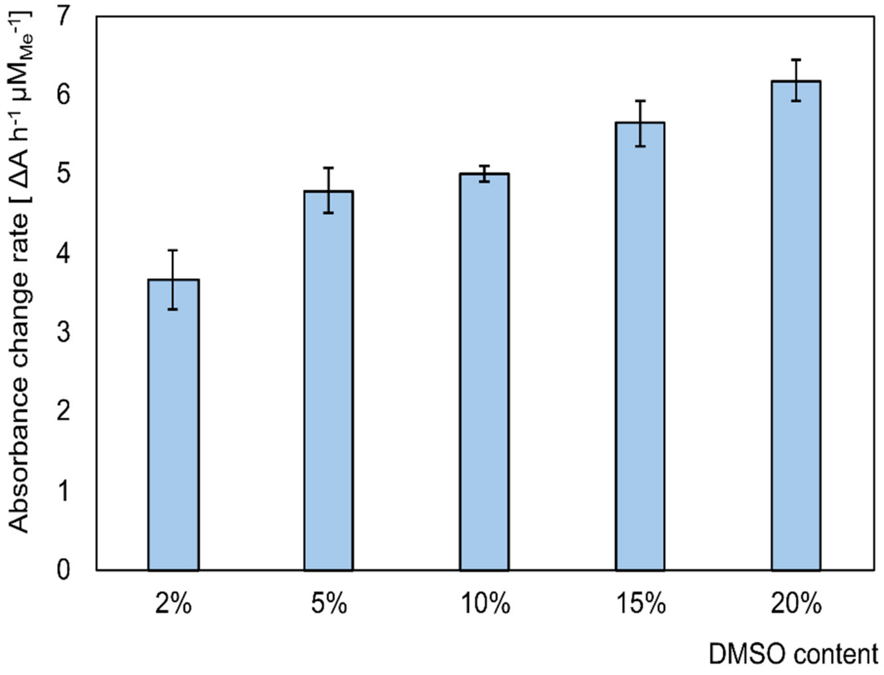

Figure S5. Effect of DMSO concentration (2–20%, v/v) on the catalytic activity of nanozymes. Au:Pd nanoparticles (1:5, 49 kDa PVA) were used as a model catalytic system. Error bars represent the standard deviation of three independent measurements (n = 3).

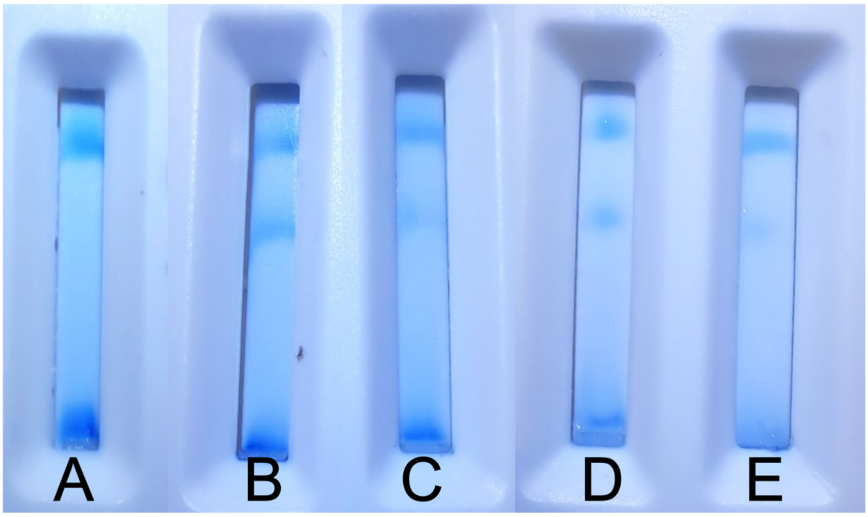

Figure S6. Oxidation-based lateral flow assay for ferritin detection employing DEPDA+NSA as chromogenic signal-generating substrates. Strip A: negative control; Strips B–E: positive control (200 ng mL^{-1} ferritin).

Table S1. Hydrodynamic diameter of various nanoparticles based on DLS measurements.

Nanoparticle type	Stabilizing agent	Hydrodynamic diameter			Mean	Standard deviation
**Au**	PVA 18 kDa	7.9	9.1	9.2	8.7	0.7
**Pt**		8.7	7.9	8.2	8.3	0.4
**Pd**		6.7	7.1	5.9	6.6	0.6
**Au:Pd 1:1**		6.4	5.6	5.9	6.0	0.4
**Au:Pd 1:5**		5.4	8.2	9.0	7.6	1.9
**Au:Pd 5:1**		7.4	7.1	7.0	7.2	0.2
**Pt:Pd 1:1**		5.0	5.7	5.7	5.5	0.4
**Pt:Pd 1:5**		5.6	5.7	5.4	5.6	0.2
**Pt:Pd 5:1**		6.4	6.9	7.5	6.9	0.5
**Au**	PVA 22 kDa	7.8	8.1	7.9	7.9	0.1
**Pt**		10.4	10.4	10.2	10.3	0.1
**Pd**		6.8	6.8	5.3	6.3	0.9
**Au:Pd 1:1**		5.2	4.7	4.9	4.9	0.3
**Au:Pd 1:5**		6.0	6.3	6.4	6.2	0.2
**Au:Pd 5:1**		6.8	6.4	6.5	6.6	0.2
**Pt:Pd 1:1**		6.6	6.9	6.8	6.8	0.1
**Pt:Pd 1:5**		6.2	6.0	5.9	6.0	0.2
**Pt:Pd 5:1**		11.2	11.7	11.4	11.4	0.2
**Au**	PVA 61 kDa	10.4	10.6	10.2	10.4	0.2
**Pt**		8.8	9.1	9.7	9.2	0.5
**Pd**		7.3	6.9	5.5	6.6	0.9
**Au:Pd 1:1**		5.5	5.6	6.4	5.8	0.5
**Au:Pd 1:5**		6.4	6.4	6.8	6.6	0.2
**Au:Pd 5:1**		7.7	8.2	7.3	7.7	0.4
**Pt:Pd 1:1**		6.7	5.3	6.0	6.0	0.7
**Pt:Pd 1:5**		6.2	5.4	5.8	5.8	0.4
**Pt:Pd 5:1**		6.1	7.7	7.3	7.0	0.9
**Au**	PVA 49 kDa	10.9	10.2	10.0	10.4	0.5
**Pt**		9.3	9.3	9.1	9.3	0.1

**Pd**		6.7	6.6	6.9	6.7	0.2
**Au:Pd 1:1**		5.1	5.0	6.8	5.6	1.0
**Au:Pd 1:5**		6.5	6.4	6.6	6.5	0.1
**Au:Pd 5:1**		7.6	6.8	7.1	7.1	0.4
**Pt:Pd 1:1**		5.7	5.6	5.7	5.7	0.1
**Pt:Pd 1:5**		5.8	6.3	6.6	6.3	0.4
**Pt:Pd 5:1**		5.7	6.6	6.9	6.4	0.6

Table S2. The values of slope of linear functions fitted to the experimental data based on the absorbance decrease in case of 4-NP substrate reduction.

**Nanoparticle type**	**Stabilizing agent**	**k - absorbance change [h^{-1}]**					**Mean**	**Standard deviation**
**Au**	PVA 18 kDa	−28.0	−33.4	−33.5	−33.9	−37.2	−33.2	3.3
**Pt**		−0.2	−0.1	−0.7	−0.4	−0.9	−0.4	0.3
**Pd**		−27.1	−26.0	−28.6	−30.2	−34.5	−29.3	3.3
**Au:Pd 1:1**		−21.8	−20.9	−22.9	−23.3	−23.4	−22.4	1.1
**Au:Pd 1:5**		−25.3	−23.9	−25.1	−27.8	−25.4	−25.5	1.4
**Au:Pd 5:1**		−18.9	−21.5	−19.4	−20.5	−22.3	−20.5	1.4
**Pt:Pd 1:1**		−17.4	−16.2	−17.9	−16.9	−21.0	−17.9	1.9
**Pt:Pd 1:5**		−24.6	−26.0	−25.7	−29.6	−29.3	−27.0	2.3
**Pt:Pd 5:1**		−4.0	−2.5	−3.9	−4.7	−7.7	−4.6	1.9
**Au**	PVA 22 kDa	−30.0	−34.5	−30.6	−33.9	−33.5	−32.5	2.1
**Pt**		−2.3	−1.9	−1.0	−1.6	−2.0	−1.8	0.5
**Pd**		−39.2	−43.3	−43.7	−46.5	−45.9	−43.7	2.9
**Au:Pd 1:1**		−28.8	−28.0	−31.3	−29.2	−26.9	−28.8	1.6
**Au:Pd 1:5**		−25.5	−26.5	−27.3	−26.0	−26.2	−26.3	0.6
**Au:Pd 5:1**		−34.1	−31.3	−35.2	−38.5	−39.0	−35.6	3.2
**Pt:Pd 1:1**		−11.1	−13.3	−12.2	−12.1	−12.0	−12.1	0.8
**Pt:Pd 1:5**		−23.1	−28.1	−25.9	−25.7	−27.7	−26.1	2.0
**Pt:Pd 5:1**		−11.5	−12.2	−11.2	−12.1	−10.8	−11.5	0.6
**Au**	PVA 61 kDa	−21.3	−18.6	−21.4	−21.4	−20.0	−20.5	1.3
**Pt**		0.2	0.5	0.5	−0.3	0.9	0.4	0.5
**Pd**		−19.6	−18.5	−21.3	−23.1	−24.6	−21.4	2.5
**Au:Pd 1:1**		−22.9	−25.8	−25.2	−25.2	−26.2	−25.1	1.3
**Au:Pd 1:5**		−22.4	−25.0	−27.1	−25.3	−30.1	−26.0	2.9
**Au:Pd 5:1**		−25.3	−27.4	−30.2	−30.6	−26.5	−28.0	2.3
**Pt:Pd 1:1**		−22.8	−21.0	−21.6	−22.9	−24.2	−22.5	1.2
**Pt:Pd 1:5**		−32.0	−31.2	−34.2	−35.0	−37.6	−34.0	2.5
**Pt:Pd 5:1**		−3.0	−3.2	−4.2	−3.5	−3.7	−3.5	0.5
**Au**	PVA 49 kDa	−38.1	−39.0	−39.5	−32.1	−36.9	−37.1	3.0
**Pt**		−1.1	0.4	−1.1	0.1	1.3	−0.1	1.0
**Pd**		−32.5	−30.8	−31.2	−30.5	−37.0	−32.4	2.7
**Au:Pd 1:1**		−28.6	−31.5	−31.8	−30.3	−33.3	−31.1	1.7
**Au:Pd 1:5**		−25.4	−26.5	−28.4	−32.3	−27.8	−28.1	2.6
**Au:Pd 5:1**		−27.6	−29.0	−29.3	−30.8	−29.7	−29.3	1.2
**Pt:Pd 1:1**		−15.4	−18.5	−19.8	−20.5	−18.2	−18.5	2.0
**Pt:Pd 1:5**		−39.1	−42.9	−43.7	−45.7	−46.4	−43.6	2.9
**Pt:Pd 5:1**		−4.3	−4.8	−4.4	−5.6	−3.1	−4.5	0.9

Table S3. The values of the slope of linear functions fitted to the experimental data based on the absorbance increase for MTT substrate reduction.

Nanoparticle type	Stabilizing agent	k – absorbance change [h^{-1}]					Mean	Standard deviation
**Au**	PVA 18 kDa	2.9	2.4	2.8	3.0	3.4	2.9	0.4
**Pt**		5.6	5.2	6.6	6.6	5.9	6.0	0.7
**Pd**		4.8	4.5	5.1	5.3	5.7	5.1	0.5
**Au:Pd 1:1**		7.2	7.1	7.4	7.4	7.7	7.3	0.2
**Au:Pd 1:5**		3.9	5.2	4.2	4.1	4.0	4.3	0.5
**Au:Pd 5:1**		4.9	6.3	5.9	6.9	6.5	6.1	0.7
**Pt:Pd 1:1**		2.2	2.7	2.0	2.4	2.3	2.3	0.3
**Pt:Pd 1:5**		5.6	5.2	5.9	6.7	6.9	6.0	0.7
**Pt:Pd 5:1**		5.4	5.3	5.5	6.0	6.1	5.7	0.4
**Au**	PVA 22 kDa	2.4	3.7	3.3	3.2	3.2	3.2	0.5
**Pt**		3.6	4.3	3.7	4.2	4.1	4.0	0.3
**Pd**		5.1	4.6	6.6	6.1	5.5	5.6	0.9
**Au:Pd 1:1**		7.1	6.8	6.8	7.5	6.4	6.9	0.4
**Au:Pd 1:5**		6.3	6.0	6.8	7.2	7.1	6.7	0.5
**Au:Pd 5:1**		4.1	5.4	4.7	4.7	4.5	4.7	0.4
**Pt:Pd 1:1**		6.3	6.4	6.3	7.9	7.5	6.9	0.7
**Pt:Pd 1:5**		6.2	7.1	6.9	8.1	7.3	7.1	0.7
**Pt:Pd 5:1**		5.4	5.2	6.1	5.4	6.2	5.7	0.5
**Au**	PVA 61 kDa	3.8	4.7	4.8	5.8	5.3	4.9	0.9
**Pt**		5.1	5.5	5.9	6.6	7.2	6.1	0.9
**Pd**		5.2	6.0	6.1	6.9	6.3	6.1	0.7
**Au:Pd 1:1**		3.9	5.4	4.0	4.5	5.1	4.6	0.8
**Au:Pd 1:5**		4.1	4.8	4.1	5.9	5.1	4.8	0.9
**Au:Pd 5:1**		8.5	8.7	9.1	9.5	10.3	9.2	0.7
**Pt:Pd 1:1**		11.4	11.4	13.0	13.2	13.8	12.6	1.1
**Pt:Pd 1:5**		7.4	7.8	8.1	8.6	8.5	8.1	0.5
**Pt:Pd 5:1**		8.7	8.6	9.2	9.1	9.1	8.9	0.3
**Au**	PVA 49 kDa	3.9	4.3	4.8	4.9	5.0	4.6	0.5
**Pt**		6.8	7.2	7.7	7.6	8.0	7.4	0.5
**Pd**		6.5	7.3	7.0	7.4	7.7	7.2	0.4
**Au:Pd 1:1**		5.1	6.1	5.8	6.3	6.8	6.0	0.6
**Au:Pd 1:5**		5.6	6.0	5.8	5.9	6.7	6.0	0.5
**Au:Pd 5:1**		8.8	10.7	9.8	10.1	10.6	10.0	0.8
**Pt:Pd 1:1**		9.6	9.4	8.8	9.8	8.9	9.4	0.5
**Pt:Pd 1:5**		6.2	6.1	6.7	6.7	7.4	6.6	0.5
**Pt:Pd 5:1**		4.7	4.8	5.8	6.3	5.3	5.4	0.8

Table S4. pH−dependent ζ−potential measurements of Au:Pd (1:1) nanoparticles.

	Mean ζ−potential [mV]	Standard deviation
**Without buffer**	−43.5	2.1
**pH 4.5**	−27.9	1.7
**pH 7.0**	−25.8	1.5
**pH 9.0**	−38.9	1.2
